# Poly[bis­(μ_2_-4,4′-bipyridine)bis­(3-nitro­benzoato)cobalt(II)]

**DOI:** 10.1107/S1600536809039786

**Published:** 2009-10-03

**Authors:** Pei-Hsuan Chiang, Shih-Chen Hsu, Chia-Her Lin

**Affiliations:** a Department of Chemistry, Chung-Yuan Christian University, Chung-Li 320, Taiwan

## Abstract

The hydro­thermal reaction of cobalt nitrate with 4,4′-bipyridine and 3-nitro­benzoic acid lead to the formation of the title complex, [Co(C_7_H_4_NO_4_)_2_(C_10_H_8_N_2_)_2_]_*n*_. In the crystal structure, the Co^II^ atoms are coordinated by two terminal carboxyl­ate anions and four 4,4′-bipyridine ligands within slightly distorted octa­hedra. The Co^II^ atom and one of the two independent 4,4′-bipyridine ligands are located on a twofold rotation axis, while the second independent 4,4′-bipyridine mol­ecule is located on a centre of inversion. One of the two rings of one 4,4′-bipyridine ligand is disordered over two orientations and was refined using a split model [occupancy ratio 0.68 (2):0.32 (2)]. The Co^II^ atoms are connected by the 4,4′-bipyridine ligands into layers, which are located parallel to the *ab* plane.

## Related literature

For background information on the solvothermal synthesis of coordination polymers with organic ligands, see: Kitagawa *et al.* (2004 ▶). For related structures, see: Biradha *et al.* (1999 ▶).
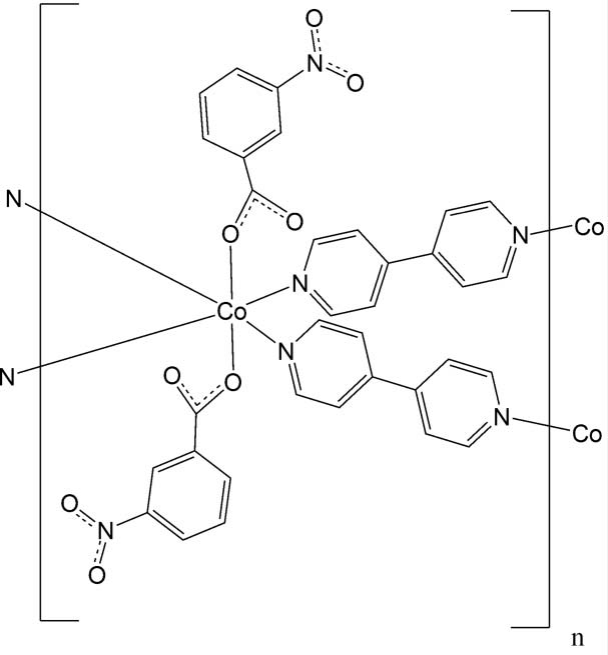


## Experimental

### 

#### Crystal data

[Co(C_7_H_4_NO_4_)_2_(C_10_H_8_N_2_)_2_]
*M* *_r_* = 703.52
Monoclinic, 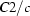

*a* = 18.2074 (15) Å
*b* = 11.4717 (8) Å
*c* = 15.0543 (12) Å
β = 94.661 (2)°
*V* = 3134.0 (4) Å^3^
*Z* = 4
Mo *K*α radiation
μ = 0.61 mm^−1^
*T* = 295 K
0.40 × 0.25 × 0.15 mm

#### Data collection

Bruker APEXII CCD diffractometer
Absorption correction: multi-scan (*SADABS*; Bruker, 2008 ▶) *T*_min_ = 0.792, *T*_max_ = 0.914
13056 measured reflections
3861 independent reflections
3389 reflections with *I* > 2σ(*I*)
*R*_int_ = 0.023

#### Refinement

*R*[*F* ^2^ > 2σ(*F* ^2^)] = 0.030
*wR*(*F* ^2^) = 0.085
*S* = 1.03
3861 reflections
243 parameters
H-atom parameters constrained
Δρ_max_ = 0.29 e Å^−3^
Δρ_min_ = −0.25 e Å^−3^

### 

Data collection: *APEX2* (Bruker, 2008 ▶); cell refinement: *SAINT* (Bruker, 2008 ▶); data reduction: *SAINT*; program(s) used to solve structure: *SHELXS97* (Sheldrick, 2008 ▶); program(s) used to refine structure: *SHELXL97* (Sheldrick, 2008 ▶); molecular graphics: *DIAMOND* (Brandenburg, 2009); software used to prepare material for publication: *SHELXL97*.

## Supplementary Material

Crystal structure: contains datablocks I, global. DOI: 10.1107/S1600536809039786/nc2159sup1.cif

Structure factors: contains datablocks I. DOI: 10.1107/S1600536809039786/nc2159Isup2.hkl

Additional supplementary materials:  crystallographic information; 3D view; checkCIF report

## Figures and Tables

**Fig. 1.**
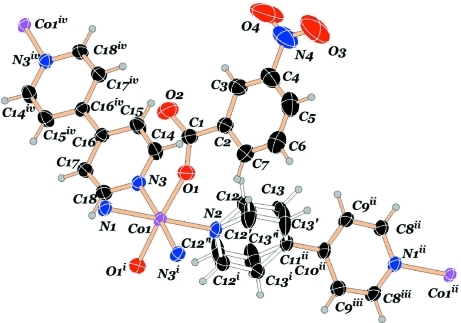
Crystal structure of the title compound with labeling and displacement ellipsoids drawn at the 50% probability level. [symmetry codes: (i) -x + 1,y,-z + 5/2; (ii) x,y - 1,z; (iii) -x + 1,y + 1,-z + 5/2; (iv) -x + 1/2,-y + 5/2,-z + 2.]. The H atoms are omitted for clarity.

**Fig. 2.**
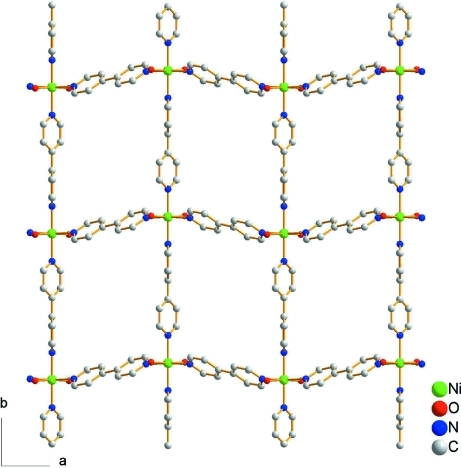
Crystal structure of the title compound in c-direction showing the layers. The H atoms and the disordered C atoms are not shown for clarity.

## supplementary crystallographic information

### Comment

The synthesis of coordination polymers has been a subject of intense research owing to their interesting structural chemistry and potential applications. A large number of these compounds have been synthesized by the reactions of metal salts and organic dicarboxyl acids or bipyridines (Kitagawa *et al.* 2004). As a further study in this field, the structure of the title compound is reported.

The asymmetric unit of the title compound consists of one Co^II^ atom, one 3-nitrobenzoate anion and two half 4,4'-bipyridine ligands (Figure 1). The octahedral metal ions are coordinated by four nitrogen atoms of two pairs of crystallographically independent 4,4'-bipyridine ligands and two oxygen atoms of two symmetry related 3-nitro benzoate anions. The Co—O bond length is 2.0557 (13) Å and the average Co—N distance amount to 2.1836 (19) Å. The metal centers are linked via the 4,4'-bipyridine ligands into layers and the anions are only terminal bonded to the Co^II^ atoms (Figure 2). Thus, this structure is different from the analogous nickel compound with the same ligands (Biradha *et al.* 1999).

### Experimental

The title compound was prepared by the reaction of 4,4'-bipyridine (0.0781 g, 0.5 mmol), 3-nitrobenzoic acid (0.0836 g, 0.5 mmol), Co(NO_3_)_2._6H_2_O (0.1454 g, 0.5 mmol), H_2_O (12.0 ml) and NH_4_OH (0.1 ml) at a pH value of 9.28. The mixture was heated to 423 K for 2 days in a Teflon-lined autoclave with an internal volume of 23 ml followed by slow cooling at 6 K/h to room temperature. The title compound was obtained as orange crystals with a yield of 0.0284 g (7.7%, based on cobalt). Anal. found/calcd.: C, 58.11/58.06; N, 12.14/11.95; H, 3.46/3.44%.

### Refinement

The hydrogen atoms of benzene rings are placed in idealized positions and constrained to ride on their parent atoms, with C—H = 0.93 Å and *U*_iso_(H) = 1.2 *U*_eq_(C). The C12 and C13 atoms are disordered and were refined using a split model with occupancies of 0.68 (2) and 0.32 (2).

### Crystal data

**Table:**

[Co(C_7_H_4_NO_4_)_2_(C_10_H_8_N_2_)_2_]	*F*(000) = 1444
*M**_r_* = 703.52	*D*_x_ = 1.491 Mg m^−^^3^
Monoclinic, *C*2/*c*	Mo *K*α radiation, λ = 0.71073 Å
Hall symbol: -C 2yc	Cell parameters from 6091 reflections
*a* = 18.2074 (15) Å	θ = 2.3–28.1°
*b* = 11.4717 (8) Å	µ = 0.61 mm^−^^1^
*c* = 15.0543 (12) Å	*T* = 295 K
β = 94.661 (2)°	Columnar, pink
*V* = 3134.0 (4) Å^3^	0.40 × 0.25 × 0.15 mm
*Z* = 4	

### Data collection

**Table:**

Bruker APEXII CCD diffractometer	3861 independent reflections
Radiation source: fine-focus sealed tube	3389 reflections with *I* > 2σ(*I*)
graphite	*R*_int_ = 0.023
Detector resolution: 8.3333 pixels mm^-1^	θ_max_ = 28.3°, θ_min_ = 2.1°
φ and ω scans	*h* = −24→23
Absorption correction: multi-scan (*SADABS*; Bruker, 2008)	*k* = −8→15
*T*_min_ = 0.792, *T*_max_ = 0.914	*l* = −19→20
13056 measured reflections	

### Refinement

**Table:**

Refinement on *F*^2^	Primary atom site location: structure-invariant direct methods
Least-squares matrix: full	Secondary atom site location: difference Fourier map
*R*[*F*^2^ > 2σ(*F*^2^)] = 0.030	Hydrogen site location: inferred from neighbouring sites
*wR*(*F*^2^) = 0.085	H-atom parameters constrained
*S* = 1.03	*w* = 1/[σ^2^(*F*_o_^2^) + (0.0451*P*)^2^ + 1.8393*P*] where *P* = (*F*_o_^2^ + 2*F*_c_^2^)/3
3861 reflections	(Δ/σ)_max_ = 0.002
243 parameters	Δρ_max_ = 0.29 e Å^−^^3^
0 restraints	Δρ_min_ = −0.25 e Å^−^^3^

### Special details

**Table:**

Geometry. All e.s.d.'s (except the e.s.d. in the dihedral angle between two l.s. planes) are estimated using the full covariance matrix. The cell e.s.d.'s are taken into account individually in the estimation of e.s.d.'s in distances, angles and torsion angles; correlations between e.s.d.'s in cell parameters are only used when they are defined by crystal symmetry. An approximate (isotropic) treatment of cell e.s.d.'s is used for estimating e.s.d.'s involving l.s. planes.
Refinement. Refinement of *F*^2^ against ALL reflections. The weighted *R*-factor *wR* and goodness of fit *S* are based on *F*^2^, conventional *R*-factors *R* are based on *F*, with *F* set to zero for negative *F*^2^. The threshold expression of *F*^2^ > σ(*F*^2^) is used only for calculating *R*-factors(gt) *etc*. and is not relevant to the choice of reflections for refinement. *R*-factors based on *F*^2^ are statistically about twice as large as those based on *F*, and *R*- factors based on ALL data will be even larger.

### Fractional atomic coordinates and isotropic or equivalent isotropic displacement parameters (Å^2^)

**Table:**

	*x*	*y*	*z*	*U*_iso_*/*U*_eq_	Occ. (<1)
Co1	0.5000	1.191582 (19)	1.2500	0.02382 (8)	
O1	0.57262 (6)	1.18588 (9)	1.15246 (7)	0.0354 (2)	
O2	0.55038 (8)	1.25715 (13)	1.01478 (8)	0.0558 (3)	
O3	0.81068 (14)	1.0206 (2)	0.83179 (16)	0.1227 (9)	
O4	0.70904 (15)	1.1028 (2)	0.79153 (13)	0.1102 (8)	
N1	0.5000	1.38167 (14)	1.2500	0.0311 (3)	
N2	0.5000	0.99916 (13)	1.2500	0.0300 (3)	
N3	0.40427 (7)	1.19830 (9)	1.15518 (8)	0.0298 (2)	
N4	0.75458 (14)	1.06877 (18)	0.84869 (16)	0.0765 (6)	
C1	0.58770 (8)	1.20319 (12)	1.07270 (10)	0.0330 (3)	
C2	0.66071 (9)	1.15062 (13)	1.05012 (10)	0.0347 (3)	
C3	0.67493 (10)	1.13700 (14)	0.96153 (11)	0.0427 (4)	
H3A	0.6406	1.1601	0.9158	0.051*	
C4	0.74128 (12)	1.08841 (15)	0.94287 (13)	0.0517 (5)	
C5	0.79520 (11)	1.05707 (17)	1.00796 (16)	0.0590 (5)	
H5A	0.8399	1.0267	0.9930	0.071*	
C6	0.78115 (11)	1.07189 (17)	1.09553 (15)	0.0548 (5)	
H6A	0.8169	1.0522	1.1407	0.066*	
C7	0.71369 (9)	1.11618 (14)	1.11679 (12)	0.0435 (4)	
H7A	0.7039	1.1229	1.1762	0.052*	
C8	0.49826 (9)	1.44210 (12)	1.17370 (10)	0.0359 (3)	
H8A	0.4975	1.4011	1.1203	0.043*	
C9	0.49748 (9)	1.56253 (12)	1.17056 (10)	0.0370 (3)	
H9A	0.4953	1.6012	1.1161	0.044*	
C10	0.5000	1.62512 (16)	1.2500	0.0317 (4)	
C11	0.5000	1.75431 (16)	1.2500	0.0314 (4)	
C12	0.5282 (4)	0.9374 (4)	1.1858 (3)	0.0383 (11)	0.68 (2)
H12	0.5490	0.9779	1.1406	0.046*	0.68 (2)
C13	0.5285 (4)	0.8176 (4)	1.1825 (3)	0.0402 (11)	0.68 (2)
H13	0.5477	0.7793	1.1351	0.048*	0.68 (2)
C12'	0.5517 (9)	0.9382 (10)	1.2151 (18)	0.060 (4)	0.32 (2)
H12'	0.5890	0.9784	1.1896	0.071*	0.32 (2)
C13'	0.5538 (10)	0.8169 (9)	1.2143 (19)	0.068 (5)	0.32 (2)
H13'	0.5920	0.7785	1.1892	0.082*	0.32 (2)
C14	0.40078 (8)	1.15400 (14)	1.07344 (10)	0.0362 (3)	
H14A	0.4399	1.1087	1.0574	0.043*	
C15	0.34177 (8)	1.17185 (14)	1.01086 (10)	0.0369 (3)	
H15A	0.3422	1.1395	0.9543	0.044*	
C16	0.28195 (7)	1.23815 (12)	1.03276 (9)	0.0281 (3)	
C17	0.28502 (9)	1.28120 (15)	1.11918 (10)	0.0386 (3)	
H17A	0.2458	1.3239	1.1381	0.046*	
C18	0.34630 (9)	1.26053 (15)	1.17697 (10)	0.0381 (3)	
H18A	0.3474	1.2915	1.2341	0.046*	

### Atomic displacement parameters (Å^2^)

**Table:**

	*U*^11^	*U*^22^	*U*^33^	*U*^12^	*U*^13^	*U*^23^
Co1	0.02889 (14)	0.01737 (12)	0.02410 (13)	0.000	−0.00451 (9)	0.000
O1	0.0408 (6)	0.0355 (5)	0.0300 (5)	−0.0014 (4)	0.0041 (4)	0.0005 (4)
O2	0.0581 (8)	0.0690 (9)	0.0398 (7)	0.0156 (7)	0.0004 (6)	0.0141 (6)
O3	0.1288 (19)	0.144 (2)	0.1065 (16)	0.0236 (16)	0.0775 (15)	−0.0159 (14)
O4	0.137 (2)	0.150 (2)	0.0493 (10)	0.0023 (16)	0.0364 (12)	−0.0075 (12)
N1	0.0420 (9)	0.0187 (7)	0.0316 (8)	0.000	−0.0024 (7)	0.000
N2	0.0347 (8)	0.0192 (7)	0.0354 (9)	0.000	−0.0011 (7)	0.000
N3	0.0314 (6)	0.0275 (6)	0.0293 (6)	0.0021 (4)	−0.0044 (5)	−0.0009 (4)
N4	0.0978 (16)	0.0672 (12)	0.0717 (13)	−0.0167 (11)	0.0522 (13)	−0.0103 (10)
C1	0.0392 (7)	0.0287 (7)	0.0307 (7)	−0.0045 (6)	0.0006 (6)	0.0000 (5)
C2	0.0435 (8)	0.0259 (6)	0.0352 (7)	−0.0049 (6)	0.0064 (6)	0.0022 (6)
C3	0.0555 (10)	0.0350 (8)	0.0389 (8)	−0.0080 (7)	0.0121 (7)	0.0018 (6)
C4	0.0668 (12)	0.0365 (8)	0.0559 (11)	−0.0120 (8)	0.0305 (9)	−0.0021 (7)
C5	0.0527 (11)	0.0418 (9)	0.0863 (15)	0.0012 (8)	0.0290 (11)	0.0038 (10)
C6	0.0469 (10)	0.0475 (10)	0.0699 (13)	0.0050 (8)	0.0040 (9)	0.0080 (9)
C7	0.0486 (9)	0.0386 (8)	0.0434 (9)	0.0020 (7)	0.0042 (7)	0.0044 (7)
C8	0.0541 (9)	0.0223 (6)	0.0304 (7)	0.0012 (6)	−0.0016 (6)	−0.0023 (5)
C9	0.0573 (9)	0.0219 (6)	0.0315 (7)	0.0014 (6)	0.0009 (6)	0.0038 (5)
C10	0.0404 (10)	0.0184 (8)	0.0361 (10)	0.000	0.0026 (8)	0.000
C11	0.0418 (10)	0.0178 (8)	0.0342 (10)	0.000	0.0010 (8)	0.000
C12	0.058 (3)	0.0214 (12)	0.0374 (18)	−0.0007 (16)	0.0131 (15)	0.0017 (11)
C13	0.063 (3)	0.0222 (13)	0.0373 (18)	0.0007 (16)	0.0180 (15)	−0.0022 (11)
C12'	0.052 (6)	0.024 (3)	0.107 (12)	−0.001 (4)	0.035 (7)	0.009 (6)
C13'	0.062 (7)	0.024 (3)	0.126 (14)	0.012 (4)	0.050 (8)	0.008 (6)
C14	0.0291 (7)	0.0399 (8)	0.0382 (8)	0.0062 (6)	−0.0050 (6)	−0.0108 (6)
C15	0.0316 (7)	0.0464 (8)	0.0315 (7)	0.0052 (6)	−0.0048 (6)	−0.0132 (6)
C16	0.0278 (6)	0.0288 (6)	0.0270 (6)	0.0008 (5)	−0.0028 (5)	0.0014 (5)
C17	0.0379 (8)	0.0490 (9)	0.0281 (7)	0.0165 (7)	−0.0030 (6)	−0.0023 (6)
C18	0.0414 (8)	0.0461 (8)	0.0254 (7)	0.0123 (7)	−0.0055 (6)	−0.0040 (6)

### Geometric parameters (Å, °)

**Table:**

Co1—O1	2.0553 (11)	C7—H7A	0.9300
Co1—O1^i^	2.0553 (11)	C8—C9	1.3824 (19)
Co1—N3	2.1627 (12)	C8—H8A	0.9300
Co1—N3^i^	2.1627 (12)	C9—C10	1.3925 (17)
Co1—N1	2.1807 (16)	C9—H9A	0.9300
Co1—N2	2.2074 (16)	C10—C9^i^	1.3925 (17)
O1—C1	1.2689 (18)	C10—C11	1.482 (3)
O2—C1	1.2283 (19)	C11—C13'^ii^	1.359 (12)
O3—N4	1.206 (3)	C11—C13'^iii^	1.359 (11)
O4—N4	1.210 (3)	C11—C13^iii^	1.384 (5)
N1—C8	1.3398 (16)	C11—C13^ii^	1.384 (5)
N1—C8^i^	1.3398 (16)	C12—C13	1.375 (7)
N2—C12'	1.315 (12)	C12—H12	0.9300
N2—C12'^i^	1.315 (12)	C13—C11^iv^	1.384 (5)
N2—C12^i^	1.334 (5)	C13—H13	0.9300
N2—C12	1.334 (5)	C12'—C13'	1.392 (16)
N3—C14	1.3280 (18)	C12'—H12'	0.9300
N3—C18	1.3370 (19)	C13'—C11^iv^	1.359 (11)
N4—C4	1.475 (3)	C13'—H13'	0.9300
C1—C2	1.523 (2)	C14—C15	1.385 (2)
C2—C3	1.388 (2)	C14—H14A	0.9300
C2—C7	1.392 (2)	C15—C16	1.390 (2)
C3—C4	1.380 (3)	C15—H15A	0.9300
C3—H3A	0.9300	C16—C17	1.388 (2)
C4—C5	1.377 (3)	C16—C16^v^	1.488 (2)
C5—C6	1.374 (3)	C17—C18	1.379 (2)
C5—H5A	0.9300	C17—H17A	0.9300
C6—C7	1.390 (2)	C18—H18A	0.9300
C6—H6A	0.9300		
			
O1—Co1—O1^i^	176.35 (6)	C5—C6—H6A	119.9
O1—Co1—N3	93.46 (5)	C7—C6—H6A	119.9
O1^i^—Co1—N3	86.68 (5)	C2—C7—C6	120.74 (17)
O1—Co1—N3^i^	86.67 (5)	C2—C7—H7A	119.6
O1^i^—Co1—N3^i^	93.46 (5)	C6—C7—H7A	119.6
N3—Co1—N3^i^	175.92 (6)	N1—C8—C9	123.08 (14)
O1—Co1—N1	91.82 (3)	N1—C8—H8A	118.5
O1^i^—Co1—N1	91.82 (3)	C9—C8—H8A	118.5
N3—Co1—N1	87.96 (3)	C8—C9—C10	119.11 (14)
N3^i^—Co1—N1	87.96 (3)	C8—C9—H9A	120.4
O1—Co1—N2	88.18 (3)	C10—C9—H9A	120.4
O1^i^—Co1—N2	88.18 (3)	C9—C10—C9^i^	117.92 (17)
N3—Co1—N2	92.04 (3)	C9—C10—C11	121.04 (9)
N3^i^—Co1—N2	92.04 (3)	C9^i^—C10—C11	121.04 (9)
N1—Co1—N2	180.000 (1)	C13'^ii^—C11—C13'^iii^	116.3 (9)
C1—O1—Co1	150.73 (10)	C13'^ii^—C11—C13^iii^	109.8 (6)
C8—N1—C8^i^	117.69 (17)	C13'^iii^—C11—C13^ii^	109.8 (6)
C8—N1—Co1	121.16 (8)	C13^iii^—C11—C13^ii^	116.7 (4)
C8^i^—N1—Co1	121.16 (8)	C13'^ii^—C11—C10	121.9 (4)
C12'—N2—C12'^i^	115.7 (10)	C13'^iii^—C11—C10	121.9 (5)
C12'—N2—C12^i^	109.7 (5)	C13^iii^—C11—C10	121.7 (2)
C12'^i^—N2—C12	109.7 (5)	C13^ii^—C11—C10	121.7 (2)
C12^i^—N2—C12	115.9 (4)	N2—C12—C13	124.0 (4)
C12'—N2—Co1	122.1 (5)	N2—C12—H12	118.0
C12'^i^—N2—Co1	122.1 (5)	C13—C12—H12	118.0
C12^i^—N2—Co1	122.1 (2)	C12—C13—C11^iv^	119.7 (4)
C12—N2—Co1	122.1 (2)	C12—C13—H13	120.2
C14—N3—C18	116.89 (12)	C11^iv^—C13—H13	120.2
C14—N3—Co1	124.98 (10)	N2—C12'—C13'	123.8 (10)
C18—N3—Co1	117.84 (9)	N2—C12'—H12'	118.1
O3—N4—O4	122.7 (2)	C13'—C12'—H12'	118.1
O3—N4—C4	118.7 (3)	C11^iv^—C13'—C12'	120.2 (9)
O4—N4—C4	118.5 (2)	C11^iv^—C13'—H13'	119.9
O2—C1—O1	126.93 (15)	C12'—C13'—H13'	119.9
O2—C1—C2	118.91 (14)	N3—C14—C15	123.34 (13)
O1—C1—C2	114.15 (13)	N3—C14—H14A	118.3
C3—C2—C7	119.26 (15)	C15—C14—H14A	118.3
C3—C2—C1	119.56 (14)	C14—C15—C16	119.93 (13)
C7—C2—C1	121.18 (14)	C14—C15—H15A	120.0
C4—C3—C2	118.41 (17)	C16—C15—H15A	120.0
C4—C3—H3A	120.8	C17—C16—C15	116.37 (12)
C2—C3—H3A	120.8	C17—C16—C16^v^	121.74 (15)
C3—C4—C5	123.09 (18)	C15—C16—C16^v^	121.89 (16)
C3—C4—N4	118.2 (2)	C18—C17—C16	119.94 (13)
C5—C4—N4	118.7 (2)	C18—C17—H17A	120.0
C6—C5—C4	118.21 (18)	C16—C17—H17A	120.0
C6—C5—H5A	120.9	N3—C18—C17	123.48 (14)
C4—C5—H5A	120.9	N3—C18—H18A	118.3
C5—C6—C7	120.21 (19)	C17—C18—H18A	118.3

Symmetry codes: (i) −*x*+1, *y*, −*z*+5/2; (ii) *x*, *y*+1, *z*; (iii) −*x*+1, *y*+1, −*z*+5/2; (iv) *x*, *y*−1, *z*; (v) −*x*+1/2, −*y*+5/2, −*z*+2.
